# A system-wide approach to digital equity: the Digital Access Coordinator program in primary care

**DOI:** 10.1093/jamia/ocae104

**Published:** 2024-05-13

**Authors:** Jorge A Rodriguez, Michelle Zelen, Jessica Szulak, Katie Moore, Lee Park

**Affiliations:** Division of General Internal Medicine and Primary Care, Brigham and Women’s Hospital, Boston, MA 02120, United States; Harvard Medical School, Boston, MA 02115, United States; Mass General Brigham, Somerville, MA 02145, United States; Mass General Brigham, Somerville, MA 02145, United States; Mass General Brigham, Somerville, MA 02145, United States; Harvard Medical School, Boston, MA 02115, United States; Mass General Brigham, Somerville, MA 02145, United States

**Keywords:** digital health, health equity, digital inclusion, health disparities, primary care

## Abstract

**Introduction:**

The transition to digital tools prompted by the pandemic made evident digital disparities. To address digital literacy gaps, we implemented a system-wide digital navigation program.

**Methods:**

The Digital Access Coordinator (DAC) program consists of 12 multilingual navigators who support patients in enrolling and using the patient portal and digital tools. We implemented the program in our primary care network which consists of 1.25 million patients across 1211 clinicians.

**Results:**

From May 2021 to November 2022, the DACs completed outreach to 16 045 patients. Of the 13 413 patients they reached, they successfully enrolled 8193 (61%) patients in the patient portal. Of those patients they enrolled, most patients were of Other race, Hispanic ethnicity, and were English-speaking (44%) and Spanish-speaking patients (44%). Using our embedded model, we increased enrollment across 7 clinics (mean increase: 21.3%, standard deviation: 9.2%). Additionally, we identified key approaches for implementing a digital navigation program.

**Conclusion:**

Organizations can support patient portal enrollment, a key part of digital health equity, by creating and prioritizing digital navigation programs.

## Introduction

The rapid transition to a digital front door of healthcare prompted by the pandemic made evident disparities in who had access to digital tools. Like other healthcare organizations, at the beginning of the pandemic, we found disparities in access to patient portals and video visits.^1^ For example, Spanish-speaking patients were 43% less likely to use video visits compared to English-speaking patients. Our organization was tasked with increasing digital access as part of broader health equity efforts. Digital disparities are driven by multiple factors including lack of internet access, devices, language-adapted platforms as well as limited digital literacy. Our organization undertook initiatives to address these factors including a device loaning program, patient portal translation, and digital literacy support. In this case report, we focus on addressing digital literacy gaps by implementing a digital navigation program to improve disparities in patient portal enrollment. Digital navigation has been identified as a potential solution for digital disparities by providing support for patients with limited digital literacy, but large-scale integration of digital navigation in the healthcare setting has been limited.^2^

The Digital Patient Experience team at Mass General Brigham (MGB) aimed to increase digital access by implementing a digital navigation program. Our goals were to develop, implement, and evaluate a system-wide digital navigation program that supported patients in enrolling in our patient portal.

## Methods

### Program description

We established the Digital Access Coordinator (DAC) program whose goal is to address gaps in digital literacy among MGB’s primary care population. MGB has 1.25 million patients across 1211 clinicians. The DACs are a team of 12 digital navigators who are multilingual and representative of the diverse backgrounds of our patients. They speak the top 6 non-English languages spoken by our patients: Spanish, Portuguese, Haitian-Creole, Russian, Cantonese/Mandarin, and Arabic.

DACs help patients enroll in our portal, which runs on Epic’s MyChart and was translated into the top 6 non-English languages our patients speak as part of an earlier initiative to provide a linguistically appropriate user interface. The translation was done by our organization. Epic supports limited translation in some languages. They did not support all needed languages that our patients speak. For those languages that they did support, it was limited, and we had additional customized content that required translation. DACs enroll patients (and/or care partners) in the portal and acquaint them with key features, such as secure messaging, medication renewals, or checking test results. They also educate patients on how to use external apps for virtual visits and remote patient monitoring, all of which integrate with the portal. To align with organizational strategy, we implemented the DAC program in our primary care clinics.

### Implementation team

We had a team structure where DACs were managed centrally but deployed locally at each clinic. This structure helped us manage the scope of DAC work, troubleshoot operational issues, and create efficient communication pathways. The team consisted of:

- 12 DACs covering MGB’s top 6 non-English languages distributed according to the linguistic needs of our patients (ie, hired more Spanish-speaking DACs since this was our most common non-English language) (12 FTEs).
- Central administrative staff
  - Medical Director (0.1 FTE)
  - Administrative Director (0.3 FTE)
  - Senior Program Manager (1 FTE)
  - Project Manager (1 FTE) for day-to-day DAC operations

### Implementation process

To establish the program, we performed a needs assessment, partnered with key stakeholders, established models for digital literacy support, developed workflows and resources for DACs, and defined hiring criteria for DACs.

#### Perform digital equity assessment across our organization

We performed a digital equity assessment to identify gaps in digital access. While we wanted to focus on multiple forms of digital access, we decided to focus on portal enrollments since it serves as the main patient-facing tool. This assessment revealed a range of needs with some clinics having low portal enrollments and others having most patients enrolled. Given the equity focus of the program, we also stratified all data by race, ethnicity, age, and language. Stratification by language was particularly important since it determined which DAC was most appropriate for each clinic.

#### Partner with key stakeholders

As part of the implementation strategy, we partnered with clinical and health information technology (HIT) leadership. We obtained buy-in from clinical leadership at each site since the deployment of the DAC program required workflow changes and staff to refer patients. This was critical early on as the role of the DAC was new to clinical teams. To maintain leadership engagement, we held monthly meetings with all sites where we provided updates and shared portal enrollment data. Collaboration with HIT leadership facilitated the building of key resources in the electronic health record (EHR), including referral orders, reports, and program analytics.

#### Establish DAC program models

Using this assessment, we developed 2 models of digital navigation: an embedded model and a central model.

- Embedded model
  - For clinics that had lower portal enrollments at baseline, the embedded model integrated an on-site DAC with the clinic staff, space, and workflows. Support was delivered to patients in-person.
- Central model
  - For clinics that had higher portal enrollments at baseline, the central model provided remote support. Patients needing support were identified via EHR referral order received from clinical teams or patient reports generated by the program. Support was delivered to patients via telephone.

Having these 2 models allowed us to extend the program across more primary care clinics. We piloted both models and were able to improve upon challenges that we found during the pilots. This allowed the program to launch at additional sites more seamlessly. As we expanded the program, we developed an implementation guide to onboard new clinics, especially those using the embedded model (Table 1).

**Table 1.** Clinic implementation guide for the embedded model.

Clinic need	Determine how many patients are not enrolled in the patient portal and what languages are most commonly spoken by patients.
Clinic staff engagement	Identify clinical champions who can motivate staff and patients. Obtain buy-in from clinic leadership, clinicians, and administrators.
Workflow	Incorporate DACs into the clinic’s daily workflow. Provide tip sheets, signage, and other informational materials. Pilot workflows and iterate based on regular feedback.
Evaluation	Maintain regular touchpoints with clinic leaders. Solicit regular feedback, particularly in the first 3-6 months after launch. Survey sites for feedback on areas for improvement and expansion opportunities. Share data on program outcomes to keep clinics informed and engaged.

#### Develop workflows and DAC resources

**Figure 1.**
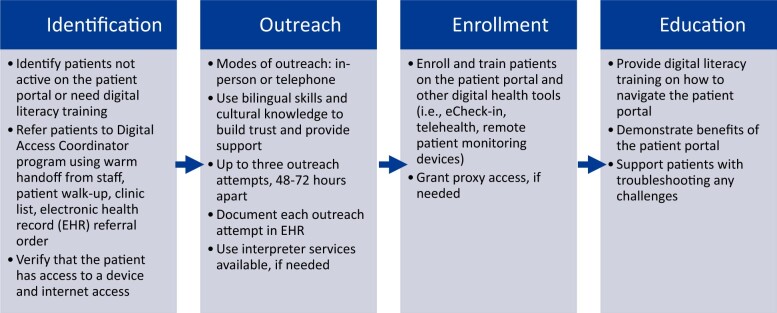
Digital access coordinator program workflow.

The introduction of DACs into the clinical team required the development of workflows that allowed DACs to interact with patients and enroll them in the portal. Overall, the DAC workflows had 4 key components: (1) identification, (2) outreach, (3) enrollment, and (4) education (Figure 1). We leveraged EHR referral orders, patient registries, worklists, and automatic documentation tools to align with these workflows and support data analytics for reporting and evaluation. For example, we deployed an “Ambulatory Referral to DAC” order that staff could submit, and it would populate a worklist that the DACs would use to follow up with patients.

We established DAC specific work areas and communication pathways. For the embedded model, we created dedicated clinic spaces where the DAC would sit and have enough space to interact with patients. Similarly, for the central model, we set up offices where the DACs could perform phone outreach. To support the DACs, we developed scripts that described the process of enrolling in the portal. Given the multilingual nature of the program, we worked with HIT teams to set up phone trees that would allow for DACs to connect patients with DACs that spoke the patient’s preferred language. There was also interpreter support available if the patient’s preferred language did not match the DAC’s spoken language. Clinics were also given postcards and flyers to advertise the program.

#### DAC hiring, onboarding, and training

Since this was a new role, we did not have clear hiring criteria or an onboarding process. We had to iterate and review external resources to determine the appropriate skill sets for a DAC.^3–5^ We prioritized DACs that spoke one of our top 6 languages. All candidates completed a language proficiency assessment in both English and their second language. Using a third-party assessment for non-English language proficiency helped ensure our DACs met the linguistic needs of our patients.^6^ DACs were expected to have some technology knowledge (ie, different devices, Microsoft Office), but not expected to be technically savvy. We were able to give DACs technical training as needed. We emphasized candidates who had strong customer service and communication skills. As the program developed, we established clear hiring criteria and could be explicit with new DACs about their expected daily work. Upon hiring, they went through a 2-week onboarding process where they received training on the portal, EHR, and workflows.

## Results

**Figure 2.**
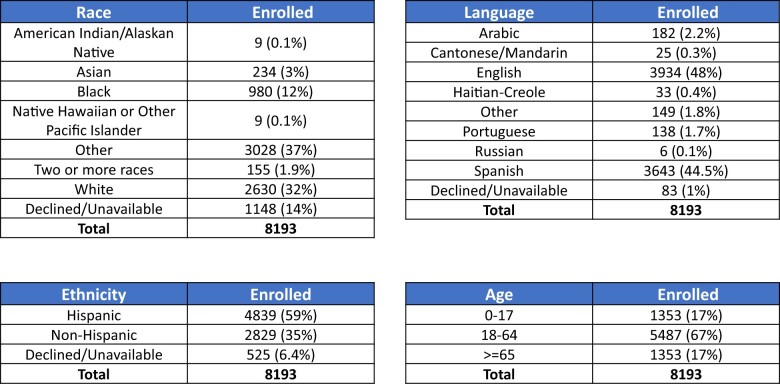
Characteristics of patient enrolled by the Digital Access Coordinator program (May 2021-November 2022).

**Figure 3.**
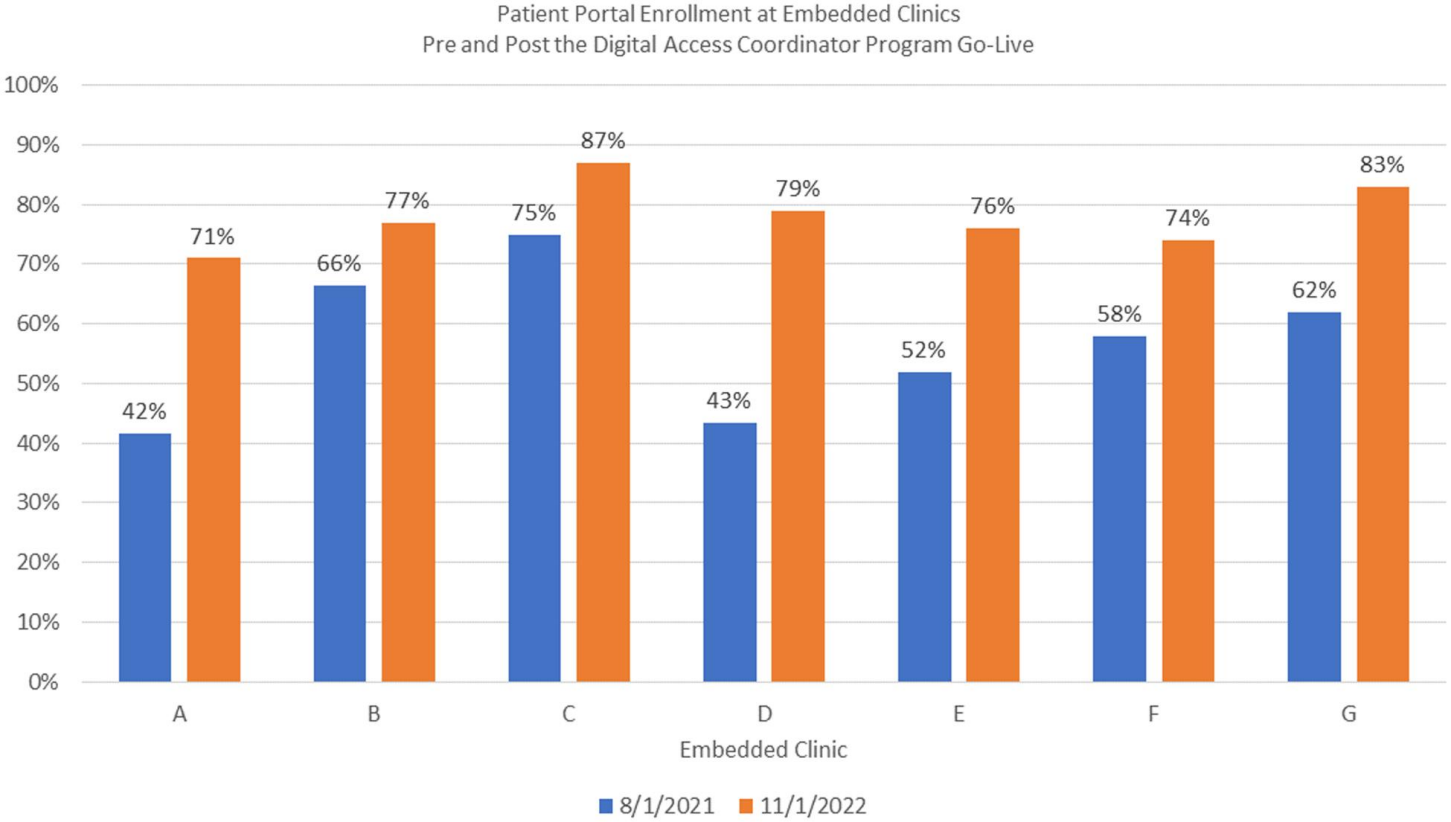
Patient portal enrolment at embedded clinics before and after the Digital Access Coordinator program.

Program evaluation relied on 2 primary metrics: outreach and portal enrollment. We tracked the total number of unique patients the DAC outreached to either in-person or via telephone call depending on the model. Of those patients that they were able to reach, we tracked their portal enrollment rate. All data were stratified by race, ethnicity, age, and language to measure equity. From May 2021 to November 2022, the DACs outreached to 16 045 patients. Of the 13 413 patients they reached, they successfully enrolled 8193 (61%) patients in the patient portal (Figure 2). Most patients were of Other race and Hispanic ethnicity. About 2854 (89%) of the patients who self-reported Other race also identified as Hispanic. We did not have more granular data on patients who identified as Other race. In terms of language, we enrolled mostly English-speaking (44%) and Spanish-speaking patients (44%). Using our embedded model, we increased portal enrollment across 7 clinics with a dedicated DAC (mean increase: 21.3%, standard deviation: 9.2%) (Figure 3). For example, from August 2021 to November 2022, clinic A increased portal enrollment from 42% before the DAC program to 74% after the program began.

Additionally, we assessed the clinic experience and patient experience. For clinic experience, we delivered a survey to 6 of our embedded clinics. Most clinics responded that the DAC program improved their ability to care for patients and clinical teams responded well to the program (Supplemental Figure S1). We also surveyed 26 patients about their experience with the program. Overall patients responded positively. They described feeling more confident about using the portal and felt that it would make it easier to manage their care.

## Program challenges

### Varied model success

The central model was more challenging to deploy. There was lower than expected referral volume. Putting in a non-clinical referral order is not a priority for clinicians, which limited the number of patients referred to the program. Additionally, supporting patients over the phone proved to be difficult. The DACs found that when “cold calling”, patients were unavailable to discuss the portal, or it was challenging to walk them through enrollment. The central model worked better for large enrollment campaign efforts which involved receiving patient lists from clinics or mailing letters followed by telephone calls.

### Data needs

Data requirements for planning and evaluation related to portal enrollment and DAC metrics required iteration. When the program went live, we initially captured program metrics by having the DACs input their activities into a shared Excel document. We did this as we were not sure what the data tracking needs would be. This introduced inaccuracies and an inability to easily track productivity and operational metrics. As we learned what documentation and tracking were necessary, we worked with HIT teams to develop EHR integrated data capture tools, which facilitated consistent reporting.

### Program funding

While prior efforts to implement digital navigation were limited by a lack of funding, we secured organizational funding for the program. The DAC program was one pillar of a larger organizational effort focused on health equity, United Against Racism.^7^

## Recommendations

For organizations implementing a digital navigation program, we would determine scope of the digital navigation, establish key digital equity metrics, and ensure stakeholder buy-in at the clinic level.

### Determine program scope and structure

Use existing digital health data (ie, portal enrollment) to identify digital disparities and determine the need for digital navigation. We suggest piloting and iterating before broadly hiring staff and expanding. Additionally, develop an operational training document for DACs to onboard, which includes scripting and portal and EHR training.

### Establish key metrics

Identify key metrics and how you will track them ahead of launch, which is ideally automated as much as possible. We suggest reviewing prior work to determine program metrics.^8^ While there is no consensus on digital access metrics, we chose outreach and enrollment as our primary metrics. As the program progresses, DACs can focus on engagement with the portal (eg, sending messages, requesting refills, having telehealth visits). Programs can use preliminary data to adjust models. For example, when the central model was not yielding consistent portal enrollment, we pivoted some DACs to other clinical sites (eg, emergency department) and models (developed a hybrid model where the DAC splits their time between being onsite and performing phone outreach).

### Maintain stakeholder engagement

Since the DACs are deployed at each clinic, but managed by a central administrative team, it is critical to delineate communication channels and roles for central leaders and local champions. We would recommend setting up a DAC program workgroup of key stakeholders to work through the implementation process and discuss issues as they arise both ahead of launch and after launching until the program is stable.

## Conclusion

The DAC program represents our organization’s focus on digital health equity as critical to our mission. The program addresses certain key factors related to digital health equity (eg, digital literacy), but does not address all multilevel drivers (eg, broadband access/affordability, clinician-level factors).^9^ Future work should focus on the implications of digital navigation on care quality and clinical outcomes and extending digital navigation to other digital tools (ie, continuous glucose monitoring, remote blood pressure monitoring). As the healthcare system becomes increasingly digital, organizations can support patient portal enrollment, a key part of digital health equity, by creating and prioritizing digital navigation programs.

## Supplementary Material

ocae104_Supplementary_Data

## Data Availability

To protect patient privacy and confidentiality, we will not be sharing individual level de-identified data. Aggregate data sets will be made available upon reasonable request.
