# Supplementary material for: A system-wide approach to digital equity: the Digital Access Coordinator program in primary care
Source: J Am Med Inform Assoc. 2024 May 13;31(7):1583–7. doi: 10.1093/jamia/ocae104 (PMC11187422; doi:10.1093/jamia/ocae104)
Supplement: ocae104_Supplementary_Data [file ocae104_supplementary_data.zip › ocae104_Supplementary_Data/DAC Patient Experience Survey_English.pdf]

# Digital Access Coordinator Patient Experience Survey

**We value your feedback and ask that you take 5 minutes to complete this brief survey on your experience with an MGB Digital Access Coordinator and the patient portal. Thank you for your time!**

1. After getting help, I feel confident about LOGGING IN to the patient portal.
    - Strongly agree
    - Agree
    - Neutral
    - Disagree
    - Strongly disagree
2. After getting help, I believe that using the patient portal will make it easier to manage my healthcare.
    - Strongly agree
    - Agree
    - Neutral
    - Disagree
    - Strongly disagree
3. I would recommend the person that helped me use Patient Gateway to others.
    - Strongly agree
    - Agree
    - Neutral
    - Disagree
    - Strongly disagree
4. I plan to use the patient portal going forward.
    - Strongly agree
    - Agree
    - Neutral
    - Disagree
    - Strongly disagree
5. Tell us about your experience working with the person that helped you use Patient Gateway. [**OPEN ENDED**]
6. If we could improve one thing about the program that helps patients use Patient Gateway, what would it be? [**OPEN ENDED**]
7. I can use applications/programs (like Zoom) on my cell phone, computer, or another electronic device on my own (without asking for help from someone else).
    - Strongly agree
    - Agree
    - Neutral
    - Disagree
    - Strongly disagree
