# Supplementary material for: A system-wide approach to digital equity: the Digital Access Coordinator program in primary care
Source: J Am Med Inform Assoc. 2024 May 13;31(7):1583–7. doi: 10.1093/jamia/ocae104 (PMC11187422; doi:10.1093/jamia/ocae104)

Supplemental Figure. Survey of Clinical Sites with an Embedded Digital Access Coordinator

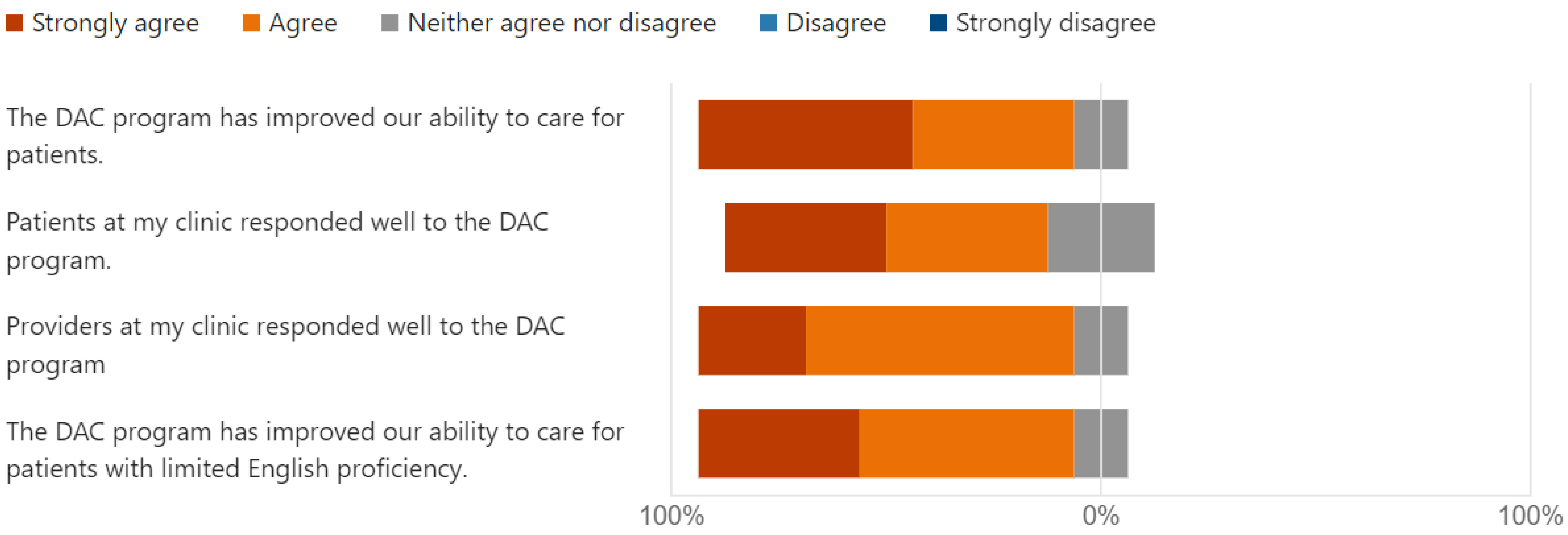

Supplement: ocae104_Supplementary_Data [file ocae104_supplementary_data.zip › ocae104_Supplementary_Data/DAC_Figure__Supplemental_Survey_REVISED.pdf]
